# Supplementary figures and images for: Mapping MAVE data for use in human genomics applications
Source: bioRxiv. 2024 Jun 30:2023.06.20.545702. Preprint. [Version 2] doi: 10.1101/2023.06.20.545702 (PMC11230167; doi:10.1101/2023.06.20.545702)

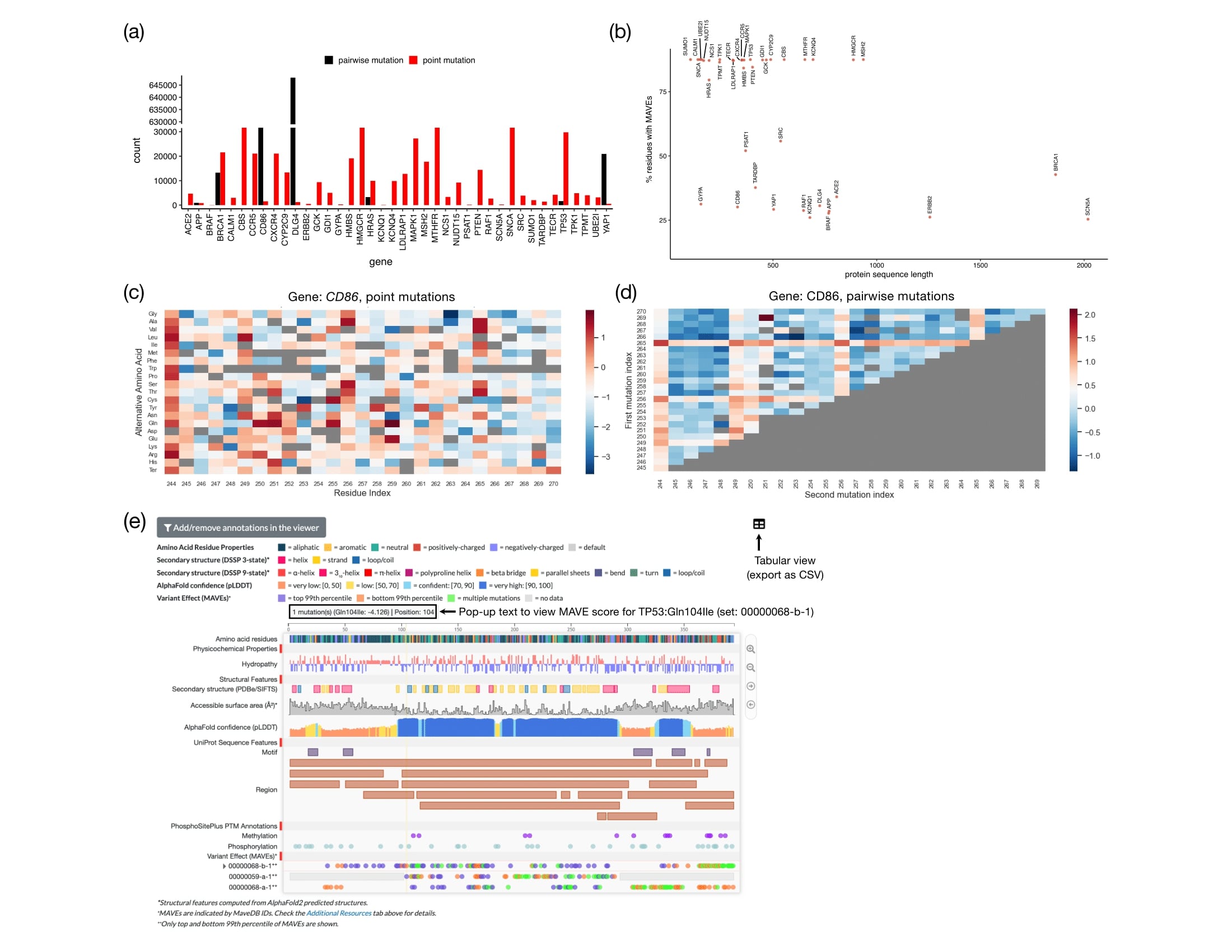

Supplement: Supplement 2 [file media-2.jpg]

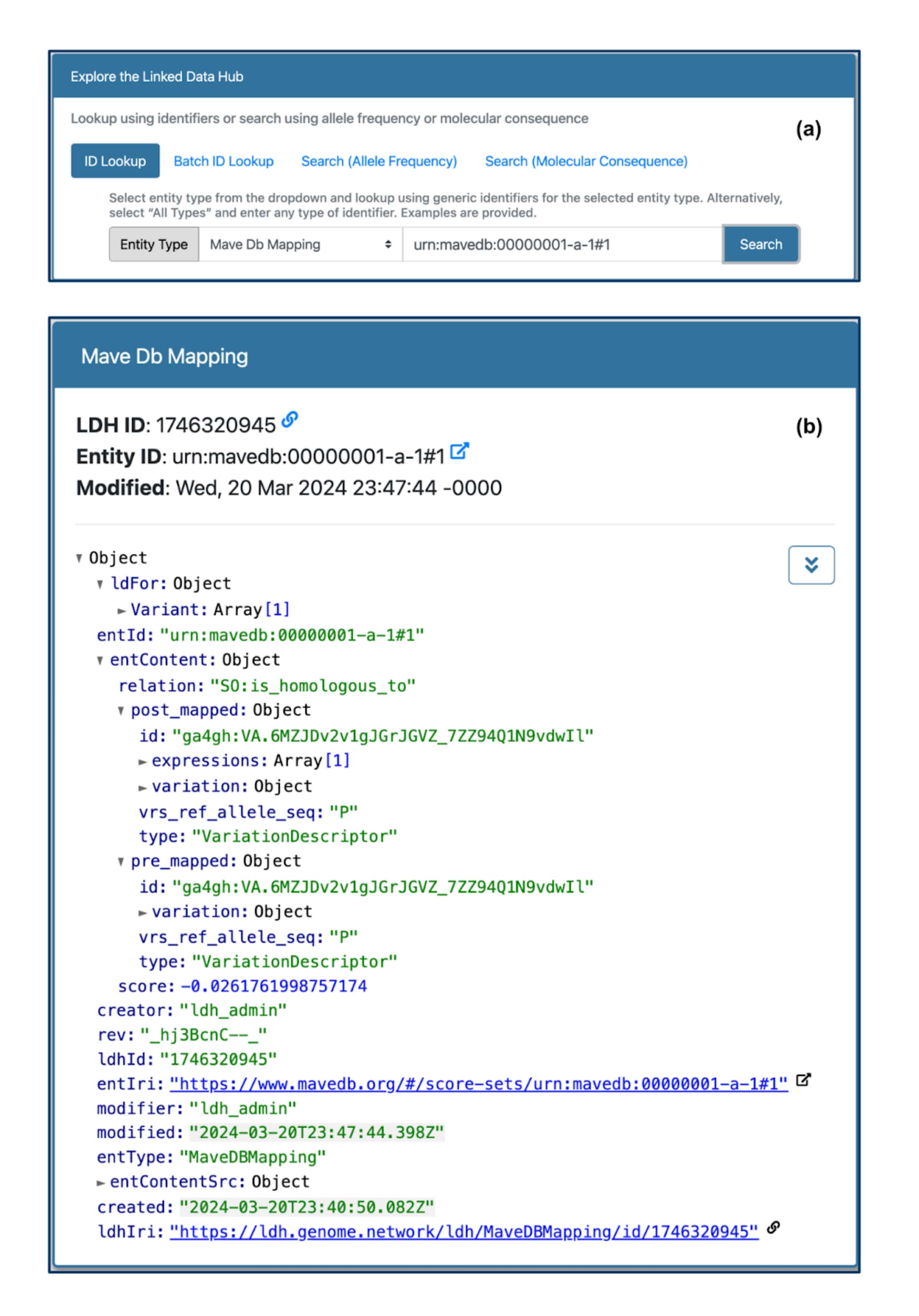

Supplement: Supplement 3 [file media-3.jpg]
